# Chronic Secondary Cardiorenal Syndrome: The Sixth Innovative Subtype

**DOI:** 10.3389/fcvm.2021.639959

**Published:** 2021-03-09

**Authors:** Yipeng Zhang, Yue Jiang, Wentao Yang, Linghong Shen, Ben He

**Affiliations:** Department of Cardiology, Shanghai Chest Hospital, Shanghai Jiao Tong University, Shanghai, China

**Keywords:** new classification, chronic secondary cardiorenal syndrome, type 6 cardiorenal syndrome, chronic co-impairment, fibrosis, biomarker

## Introduction

Heart failure, as the leading cause of cardiovascular death, has seen an increased prevalence nowadays, along with renal insufficiency. It's estimated that 25–63% of heart failure patients have the comorbidity of renal insufficiency, an independent risk factor of various cardiovascular events and predictors of poor prognosis (1). A therapeutic principle of heart failure emphasizing decongestive treatment is limited by the demand for sufficient perfusion in terms of renal insufficiency therapy, making the treatment of both concomitant diseases more complicated and contradictory, with mild balance; hence, the great interest in cardiorenal interactions has broadened among researchers and clinicians, and the concept of cardiorenal syndrome (CRS) was first proposed in 2004.

CRS, defined as a pathophysiological process of adverse interaction between the heart and kidneys, encompasses a spectrum of diseases involving acute or chronic dysfunction in one organ that induces decompensated dysfunction in the other, and eventually they evolve into an interrelated and vicious cycle of declining function in both organs. CRS was first categorized into five subtypes in 2008 based on sequential organ involvement and the course of progression over time (i.e., acute or chronic), including acute cardiorenal syndrome, chronic cardiorenal syndrome, and acute renocardiac syndrome, chronic renocardiac syndrome, secondary cardiorenal syndrome, of which a brief definition, etiology, and pathophysiology are given in Table 1 (2). With the consideration that the previous secondary CRS subtype has a shortcoming in that it is not as symmetrical as the first four subtypes, as well as the huge difference of pathophysiological changes, the treatment principles of the two organs, and of gradually increasing in-depth knowledge of fibrosis pathogenesis over the past few years, it's become quite necessary to propose a new CRS classification. And that's why we now first propose the sixth innovative CRS subtype on the basis of the concept of “chronic co-impairment” of the heart and kidneys, further classifying traditional secondary CRS as acute secondary CRS and chronic secondary CRS, thus making the novel six kinds of CRS categories paired and matched correspondingly. The most common precipitant of acute secondary CRS is biotoxin damage and a cytokine storm generated by acute sepsis, and the primary treatment principles are cause-related treatment as aggressive anti-infective therapy, as well as symptomatic treatment of cardiac and renal dysfunction. However, the novel type 6 CRS (chronic secondary CRS) is actually more prevalent in clinical practice, and its pathophysiological and clinical characteristics as well as diagnosis and treatment principles vary dramatically from other subtypes of CRS, which will be described in detail in the following article.

**Table 1:** New classification of CRS based on disease acuity and sequential organ involvement.

**Phenotype**	**Nomenclature**	**Definition**	**Common etiology**	**Pathophysiology**	**Diagnosis**	**Treatment**
Type 1 CRS	Acute cardiorenal syndrome	ACS or AHF resulting in AKI	ACS, AHF	Hemodynamic damage, RAAS activation, sympathetic neurohormonal activation		
Type 2 CRS	Chronic cardiorenal syndrome	Chronic HF resulting in CKD	Chronic HF	Chronic hypoperfusion, subclinical inflammation, accelerated atherosclerosis		
Type 3 CRS	Acute renocardiac syndrome	AKI resulting in AHF	Volume overload, inflammatory surge resulting from AKI	Hypertension, RAAS activation, sympathetic activation; electrolyte, and acid-base imbalance	Descriptive diagnosis based on history and sequential organ involvement	Respective treatment of organs involved
Type 4 CRS	Chronic renocardiac syndrome	CKD resulting in chronic HF	spectrum of CKD	Anemia, malnutrition, uremic toxins, electrolyte and coagulation imbalance, volume overload		
Type 5 CRS	Acute secondary cardiorenal syndrome	Acute systemic disease resulting in AHF and AKI/AKF	Sepsis, acute intoxication	Toxemia, systemic inflammation, cytokine storm, exogenous toxins mediated damage		
Type 6 CRS	Chronic secondary cardiorenal syndrome	Chronic systemic process resulting in HF and CKD	Diabetes mellitus, hypertension, obesity, amyloidosis, cirrhosis, systemic lupus erythematosus	Common fibrosis pathway mediated by systemic inflammation, oxidative stress, RAAS activation, sympathetic neurohormonal activation, vascular endothelial dysfunction, etc.	Comprehensive diagnosis based on definitive history, objective biomarkers (Gal-3, NGAL, ST-2) and visible imaging	Cardiorenal co-treatment represent as MRA, ARNI, SGLT-2i

*ACS, Acute Coronary Syndromes; AHF, Acute Heart Failure; AKI, Acute Kidney Injury; CKD, Chronic Kidney Disease; AKF, Acute Kidney Failure; MRA, Mineralocorticoid Receptor Antagonist; ARNI, Angiotensin Receptor Neprilysin Inhibitor; SGLT-2i, Sodium-Glucose co-Transporter-2 inhibitor*.

## Type 6 CRS

### Definition of Type 6 CRS

In clinical practice, many patients hospitalized for the first time have been examined for cardiac and renal insufficiency simultaneously, in whom it's hard to identify exactly which organ is the primary precipitant of the vicious cycle of CRS. There is the other situation that patients with chronic systematic diseases diagnosed long ago such as cirrhosis, amyloidosis, systemic lupus erythematosus, obesity, diabetes, hypertension, and hyperlipidemia may gradually evolve into declining function of both organs in the follow-up. The above two kinds of circumstances can be classified into type 6 CRS. In summary, type 6 CRS is defined as analog clinical circumstances of a clearly acknowledged onset of chronic systemic conditions in the very beginning, including diabetes, hypertension, amyloidosis, and systemic lupus, followed by gradually progressive decompensation of cardiac and renal function, culminating in cardiac and renal failure.

### Pathophysiology of Type 6 CRS

Recent studies have shown that type 6 CRS shares a common pathophysiological mechanism, i.e., that chronic systemic diseases cause systemic inflammation, renin-angiotensin-aldosterone system (RAAS) activation, sympathetic neurohormonal activation, oxidative stress, vascular endothelial dysfunction, and other pathological changes, and these finally lead to cardiac and renal fibrosis and consequent insufficiency (3, 4). There exist both overlap and difference between type 6 CRS and the five traditional cardiorenal syndromes in pathophysiological mechanisms, as illustrated in Table 1, by the detailed multiple pathophysiology of each CRS, but the common fibrosis pathway is unique to type 6 CRS with direct significance in fundamental research and clinical applications. Take hypertension, for example: it's often accompanied by RAAS activation, sympathetic nerve activation, and vascular endothelial dysfunction, on account of which increased aldosterone has been found to mediate a series of signaling cascades of fibrosis in animal models. NGAL, ST-2, and Galectin-3, as mediators of aldosterone-induced fibrosis, can promote proliferation and differentiation of fibroblasts and the secretion of extracellular matrix proteins, leading to fibrosis in both organs eventually (5). Similar pathophysiological changes can also be seen in diabetes, such as chronic systemic vascular inflammation, endothelial dysfunction, and oxidative stress, which induce increased transcription of multiple inflammatory factors. TGF-β, a well-known inflammatory factor with the broadest spectrum of effects, could affect Smad transcription factors to mediate downstream signaling pathways of fibrosis (6). Systemic lupus erythematosus (SLE) causes apoptosis and necrosis of cardiac and renal cells through direct immune injury, thus leading to an injury-related fibrosis repair process. Fibrosis is seen primarily as a protective compensation mechanism of external damage of systemic conditions, but it eventually causes chamber dilatation, heart failure, loss of nephrons, and decreased glomerular filtration rate, namely, the type 6 cardiorenal syndrome here.

### Diagnosis of Type 6 CRS

The past diagnosis of CRS was merely descriptive, on the basis of previous history, without accurate laboratory and imaging data assisting. The new CRS classification proposed here, especially the type 6 CRS, is diagnosed with the common pathogenesis pathway of fibrosis and can be differentiated in diagnosis from type 2 or type 4 CRS according to onset characteristics and complementary examinations. In addition to a patient's previous diagnostic history of chronic diseases such as hypertension, obesity, and SLE, or if the first symptoms are chronic co-impairment of cardiac and renal function without a clear precipitant, laboratory examinations can provide an important reference for diagnosis with elevated biomarkers relevant to fibrosis and other pathophysiological targets (7). Several studies have found that Gal-3, NGAL, ST-2, cardiotrophin-1, as rising indicators in a mouse fibrosis model, can be potential diagnostic biomarkers for type 6 CRS in the future (8–11). However, more evidence is needed for those markers of effective indication of fibrosis in human body. Imaging examination such as LGE-MRI has important diagnostic value for myocardial fibrosis. Kidney biopsy, as an invasive test, used to be conducted in the evaluation of the pathological type of intractable nephritis, could provide direct evidence of renal fibrosis, in which renal tubulointerstitial fibrosis, mesenchymal-epithelial transformation of interstitial cells with elevated fibroblast markers such as collagen, fibronectin, and reduced interstitial markers such as E-cadherin in immunohistochemical staining can be observed.

### Treatment of Type 6 CRS

Fibrosis is not only an indicator of diagnosis of type 6 CRS, but also the creative breakthrough in the treatment of the new subtype. Previous CRS treatments were treated, respectively, not as unified as the concept of CRS (12). Clinicians tend to treat the organ perceived to be the primary precipitant with etiological treatment and provide corresponding symptomatic support for the other organ involved in CRS, and there are often contradictory conditions, such as acute CRS, so that decongestive therapy needed for heart failure may conflict enough with renal perfusion and reflexively activate the RASS system, thus aggravating renal insufficiency, in which the inappropriate balance of the two will lead to deterioration of the CRS. The new unified therapeutic target of type 6 CRS based on the common fibrosis pathway, is mediated by RASS activation, inflammation, oxidative stress, and vascular endothelial dysfunction (13). Therefore, the treatment of type 6 CRS based on the above common target has substantial theoretical support. Current evidence shows that fibrosis is partly mediated by MR pathway (14), and mineralocorticoid receptor antagonists (MRA), as diuretics in treatment of heart failure, is an important potential anti-fibrosis drug. The RASS system inhibitors ACEI and ARB, which have been widely recognized for their positive effects on ventricular remodeling, have reduced the risk of cardiovascular events and prolonged survival, also have an anti-fibrosis effect to some degree; therefore they are expected to take a prescription for treatment of type 6 CRS. Physicians have long been quite circumspect about the usage of ACEI and ARB in HF patients with severe renal insufficiency for fear of exacerbation of renal dysfunction and hyperkalemia. Recent studies have shown that above inordinate concern can be dispelled by regular monitoring of renal function and serum potassium. A propensity score analysis of 1,665 patients with HF (EF < 45%) and eGFR <60 ml/min suggested treatment with an ACE inhibitor or ARB, which was associated with significant reductions in all-cause mortality. More clinical trials are still underway to provide evidence to confirm the clinical benefits of ACEI or ARB in patients with advanced CKD. In the future, target excavation and development of new drugs related to inflammation, endothelial dysfunction, oxidative stress, and other pathophysiological changes in CRS are of great value and prospect (15). ARNI and SGLT-2i have shown tremendous benefits to both the heart and kidneys in patients with chronic diseases, which especially demonstrates the pioneering concept of cardiorenal co-treatment (16, 17). As for the initial precipitant of the vicious circle of type 6 CRS, chronic systemic diseases should be well-controlled with respect to indispensability, such as hypertension, diabetes mellitus, obesity, etc.

## Conclusion

The sixth innovative CRS subtype, named chronic secondary CRS, is a new concept derived from the five classical CRS, and its most important value lies in its subversive notion of the diagnosis and treatment of CRS. The past CRS classification proposed in 2008 is a descriptive diagnosis based on a medical history with no specifically derived therapeutic interventions; therefore, it exerts little influence over treatment in current clinical practice. While our new type 6 CRS is based—except for disease acuity and sequential organ involvement—on detectable and visualized markers of cardiac injury including BNP and myocardial enzyme spectrum, renal function markers such as creatinine, Cys-C, and key pathophysiological markers of fibrosis of cardiorenal chronic co-impairment, together with imaging and pathological examination to make an integrated diagnosis, for which our novel pragmatic CRS categories facilitate a differentiation diagnosis from various subtypes and streamline inclusion criteria for future clinical trials. Moreover, our brand-new concept can also guide the treatment of type 6 CRS, based on the common pathogenesis of fibrosis. Unlike previous CRS for which heart and kidney dysfunction are treated separately, anti-fibrosis therapy truly realizes a comprehensive and unitary treatment based on the concept of CRS. However, there are still many fields of the new subtype worth further exploration. In addition to fibrosis, the problems of inflammation, endothelial dysfunction, oxidative stress, and other unified pathophysiological mechanisms of cardiorenalco-impairment of type 6 CRS deserve further investigation, as well as the establishment of diagnostic markers based on common pathogenesis, research for key targets, and development of corresponding potential new drugs (18). Furthermore, nowadays many large-scale RCT of drugs often exclude advanced CKD from the population included due to the concern of renal insufficiency affecting pharmacokinetics, which makes the clinical research data for excavating potential drugs specific to CRS less available. It is firmly believed that, as clinicians attach more importance to the novel concept of type 6 CRS, more cross-specialty cooperation and clinical trials for precise diagnosis and treatment of type 6 CRS will spring up constantly.

## Author Contributions

BH proposed this new classification concept and revised the manuscript. YZ wrote the manuscript. YJ, WY, and LS participated in discussions and provided useful suggestions to the conceptualization of the work. All authors contributed to the article and approved the submitted version.

## Conflict of Interest

The authors declare that the research was conducted in the absence of any commercial or financial relationships that could be construed as a potential conflict of interest.
